# Genetic Diversity of the Species of the Genus *Deschampsia* P.Beauv. (Poaceae) Based on the Analysis of the ITS Region: Polymorphism Proves Distant Hybridization

**DOI:** 10.3390/ijms252111348

**Published:** 2024-10-22

**Authors:** Alexander A. Gnutikov, Nikolai N. Nosov, Olga V. Muravenko, Alexandra V. Amosova, Victoria S. Shneyer, Igor G. Loskutov, Elizaveta O. Punina, Alexander V. Rodionov

**Affiliations:** 1 N.I. Vavilov Institute of Plant Genetic Resources (VIR), 190000 St. Petersburg, Russiai.loskutov@vir.nw.ru (I.G.L.); 2 Komarov Botanical Institute of the Russian Academy of Sciences, 197376 St. Petersburg, Russia; 3 Engelhardt Institute of Molecular Biology of RAS, 119991 Moscow, Russia

**Keywords:** *Avenella*, introgression, NGS, phylogeny, Poeae

## Abstract

The species of the genus *Deschampsia* are difficult for identification, and the genus is difficult for taxonomic treatment. The regions of 35S rRNA genes were studied for the species of the genus *Deschampsia* of different geographical origin with a method of sequencing by Sanger (ITS1–5.8S rRNA gene–ITS2, 14 species) and with a method of a locus-specific next-generation sequencing (NGS) on the Illumina platform (ITS1–5.8S rRNA, 7 species). All species of *Deschampsia* formed one clade; the species, referred by some authors on the basis of morphological characters to the species *D. cespitosa* s.l., entered one subclade. Subantarctic species formed a separate subclade and their ribotypes formed their own subnetwork. *Avenella flexuosa*, earlier referred to *Deschampsia*, entered the other clade, though this species contains some ribotypes common with some *Deschampsia* species. *Deschampsia pamirica* and related mountain species have their own specific ribotype groups. On the network of the ribotypes, one can see that *D. cespitosa* from Great Britain forms a network with some species, but *D. cespitosa* from the USA forms its own network. Ribotype analysis of each sample revealed traces of introgression with *Deyeuxia/Calamagrostis* in *D. cespitosa* and with *A. flexuosa* and probable introgression of Northern and subantarctic species.

## 1. Introduction

The genus *Deschampsia* P.Beauv. (Tussock Grass) is one of the few taxa of grasses (Poaceae Barnhart) which probably originated from intertribal hybridization processes. The total amount of the genus is about 60 species, growing in almost all extratropical areas of both hemispheres, as well as in the highlands of the tropics [1]. Originally, *Deschampsia* was referred to the tribe Aveneae Dumort. according to the morphological features of the spikelets [2,3]. Molecular phylogenetic data obtained on chloroplast genes showed that on the maternal line the taxa of this subtribe, including *Deschampsia* they are close to the genus *Festuca* L. from the subtribe Loliinae Dumort., tribe Poeae R.Br. s. str. (“Poeae chloroplast type” [4,5,6]). Based on nuclear genes, they are grouped together with the tribe Aveneae [4,7]. Because of the controversial position of the subtribe Airinae on the border of the former tribes Aveneae and Poeae R.J., Soreng proposed to combine them into one large tribe Poeae s. l. [4]. This view is now generally accepted, although the main groups of genera in the former tribes Aveneae and Poeae are quite distinct [6]. Presumably, the subtribe Airinae is hybridogenous [4,6,7]. The genus *Avenella* Bluff ex Drejer, which was sometimes considered synonymous with *Deschampsia* [2,8], is adjacent to the genus *Deschampsia*. According to modern data, *Deschampsia* is also related to the genera *Avenula* (Dumort.) Dumort. and *Helictochloa* Romero Zarco (formerly *Helictotrichon* Besser s. l.). The genus *Deschampsia* is distinguished by the chromosomal number 2n = 26, which is unusual for grasses of the *Aveneae* and *Poeae* tribes [9,10,11].

Many species belonging to the genus *Deschampsia* are characterized by extreme polymorphism, and species boundaries in it are currently the subject of debate. The complete absence of consistent characters and the insignificance of differences between species allowed some authors to recognize different species of the genus as subspecies or even varieties [8,12]. Three species of the genus, *D. borealis* (Trautv.) Roshev., *D. brevifolia* R.Br., and *D. alpina* Roem. & Schult., are high Arctic, reaching the northern limit of vegetation, and the Antarctic species—*D. antarctica* E.Desv.—is one of the grass species currently known from Antarctica [8]. Almost all Arctic species of *Deschampsia* are closely related and often hybridize with each other, apparently producing fertile hybrids [8]. The consequence of this is the abundance of populations and specimens more or less intermediate between individual species, which significantly complicates their identification [8,12]. Sometimes some authors included Arctic *Deschampsia* species in the complex *D. cespitosa* s. l. [3,12], but most likely these species had their own centers of origin during the Alpine orogeny [13], and possibly, they are also hybridogenous. *D. cespitosa* s. l. complex is characterized by an almost worldwide range and presents multiple evolutionary lines partly of which are relict according to modern molecular phylogenetic data [14].

To clarify the picture of the relationships of some representatives of the genus *Deschampsia*, we performed a molecular phylogenetic analysis of ITS1–5.8S rDNA–ITS2 sequences obtained by the Sanger method and 18SrDNA–ITS1–5.8S rDNA sequences obtained by next-generation sequencing (NGS). We need to say that Russian representatives of the genus *Deschampsia* have not been studied previously by molecular phylogenetic methods. Previous molecular phylogenetic works on *Deschampsia* species used the sequences obtained only by the Sanger method; due to hybridization. The results of these analyses did not provide a detailed resolution on the trees [15,16,17,18]. Analysis of the sequences obtained by NGS can reveal hidden events of the introgressive hybridization even when morphological features are rather constant [19].

## 2. Results

Our alignment of the ITS1–5.8S rDNA–ITS2 marker region has 609 positions in total. We used *Triticum monococcum* subsp. *aegilopoides* (Link) Thell. as an outgroup. Table 1 demonstrates information about the samples used for analysis by the Sanger and NGS methods.

**Figure 1:**
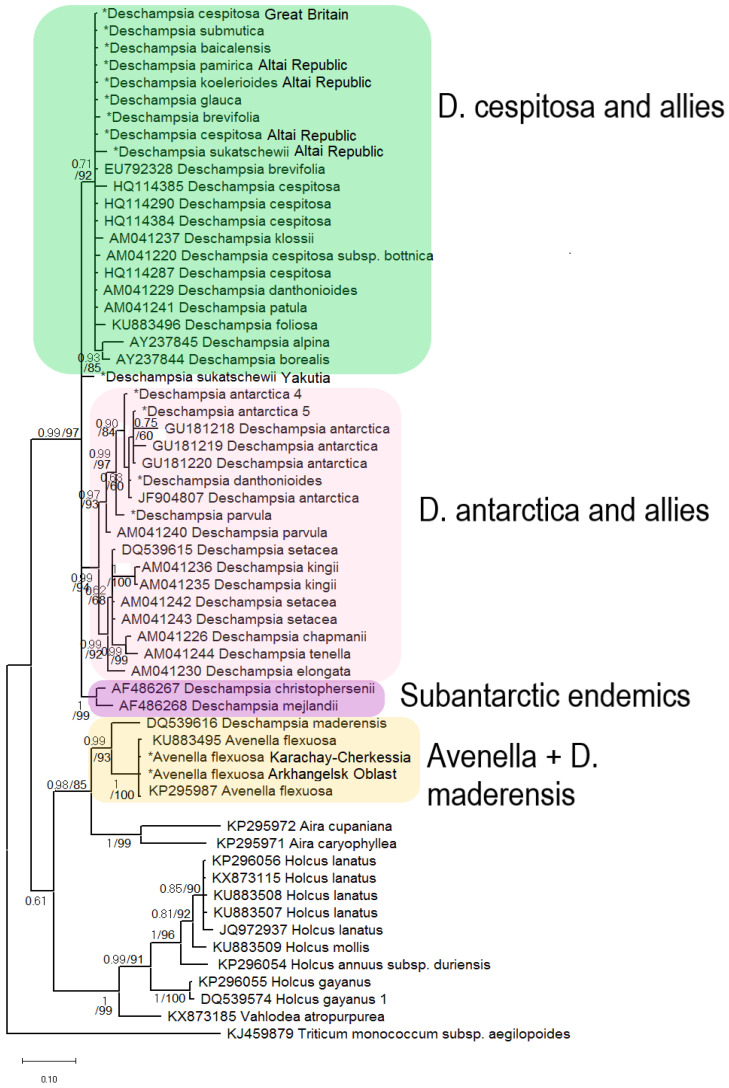
Phylogenetic tree of the studied species of subtribe Airinae according to the ITS sequence data obtained by the Sanger method. The first index on the branch is the posterior probability in Bayesian inference, the second is the bootstrap index obtained by Maximum Likelihood algorithm. When only one index is shown on the branch, it is the posterior probability. Sequences obtained by us are marked by an asterisk.

**Figure 2:**
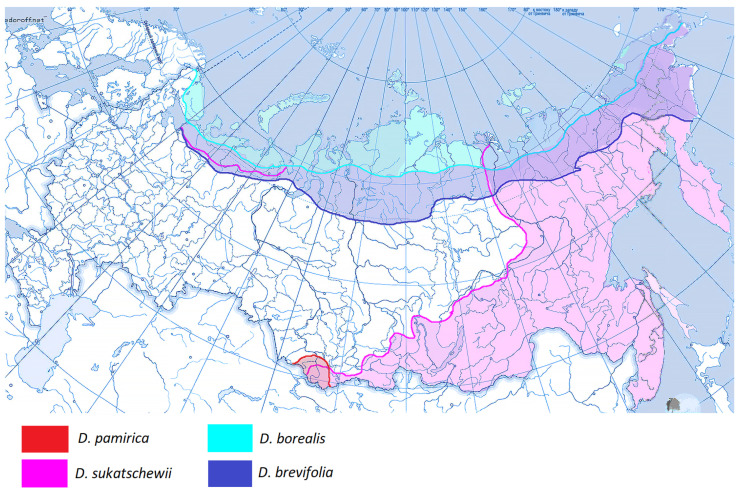
Area of distribution of the studied *Deschampsia* species in Russia (complex *D. cespitosa* s. l.). Part 1.

**Figure 3:**
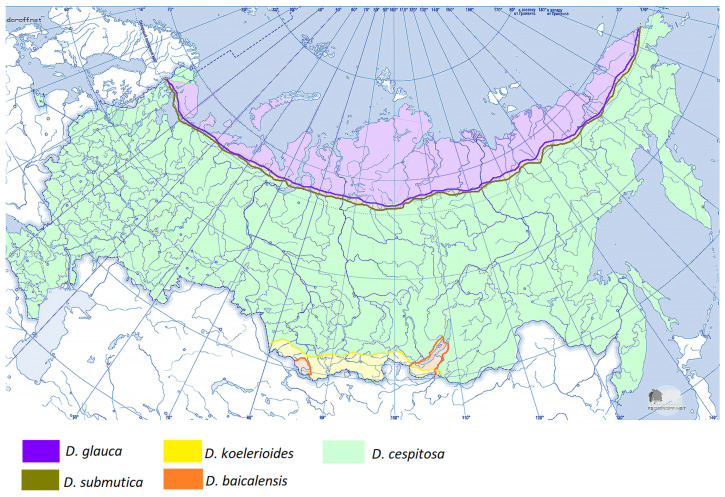
Area of distribution of the studied *Deschampsia* species in Russia (complex *D. cespitosa* s. l.). Part 2.

**Figure 4:**
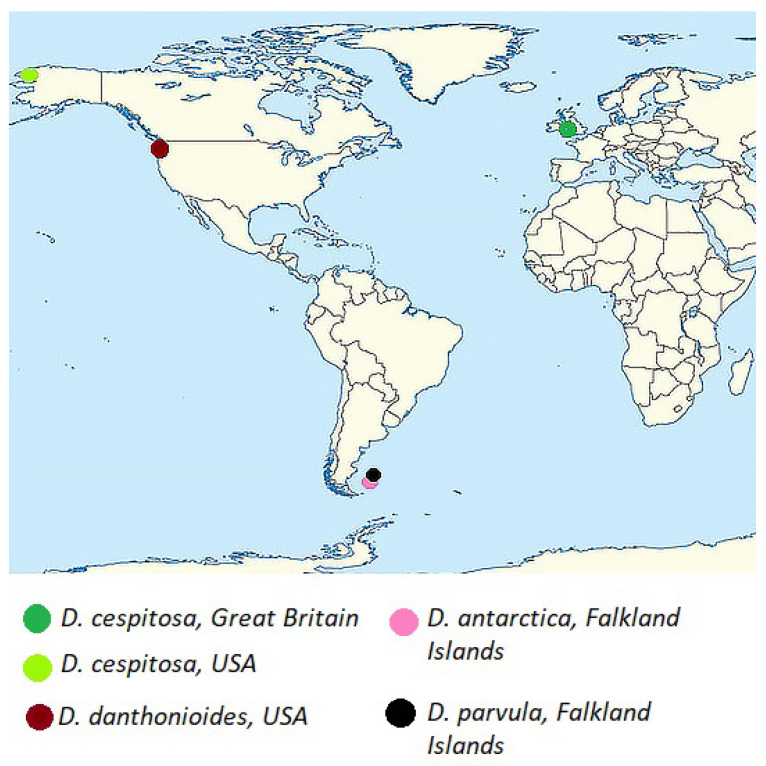
Sites of the studied samples of subantarctic *Deschampsia* species, *D. danthonioides*, and *D. cespitosa* (from Great Britain and the USA).

Members of the subtribe Airinae included in our analysis were divided into two large clades (Figure 1). The first clade corresponds to the genus *Deschampsia* (PP = 0.99, BS = 97). The second clade contains two subclades: *Avenella + Aira* (PP = 0.98, BS = 85) and *Holcus + Vahlodea* (PP = 1, BS = 91). Within the large *Deschampsia* clade, we see an interesting division into groups of species based on their geographic distribution (Figure 2, Figure 3 and Figure 4).

The clade consists of three subclades corresponding to (1) the affinity groups of *D. cespitosa* (PP = 0.71, BS = 92), (2) *D. antarctica* (PP = 0.99, BS = 94), and (3) subclade containing subantarctic endemics *D. christophersenii* C.E.Hubb. + *D. mejlandii* C.E.Hubb. (PP = 1, BS = 99). The Yakut specimen of *Deschampsia sukatschewii* occupies an uncertain position within the large *Deschampsia* clade. Subclade *Deschampsia cespitosa* and related species include *D. cespitosa* from Great Britain, *D. cespitosa* from Altai, *D. koelerioides* Regel, *D. pamirica* s. l., *D. glauca* s. l., *D. sukatschewii* s. l. (all species are from Altai Republic), *D. submutica*, *D. baicalensis* Tzvelev, *D. brevifolia*, as well as sequences from the Genbank database of *D. cespitosa* from Canada, tropical *D. klossii* Ridl. from New Guinea, *D. danthonioides* Munro from Chile, *D. patula* (Phil.) Pilg. ex Skottsb. from Argentina, and Azorean *D. foliosa* Hack. (= *Avenella foliosa* (Hack.) Rivas Mart., Lousã, Fern.Prieto, E.Días, J.C.Costa & C.Aguiar). We also observe here a separate small subclade from the Norwegian samples of *D. borealis* and *D. alpina* (PP = 0.93, BS = 85, sequences from Genbank). 

The second subclade within the large *Deschampsia* clade consists of *D. antarctica* and related species. Our *D. antarctica* specimens from the Falkland Islands, as well as *D. danthonioides* from the USA, form their own subgroup together with the *D. antarctica* sequences from GenBank (PP = 0.90, BS = 84). *Deschampsia parvula* E.Desv. (from the Falkland Islands) is a sister to this subgroup (PP = 0.99, BS = 97). The second subgroup in the *D. antarctica* subclade (PP = 0.99, BS = 92) consists of *D. setacea* (Huds.) Hack. (subarctic species, specimens from Norway), subantarctic *D. kingii* E.Desv. from Argentina (Tierra del Fuego), *D. chapmanii* Petrie from New Zealand, *D. tenella* Petrie from New Zealand; *D. elongata* (Hook.) Munro in Benth. from Argentina, Patagonia (sequences from GenBank).

All sequences of *Avenella flexuosa* (L.) Drejer do not differ significantly from each other. But close to the *A. flexuosa* specimens, it turns out to be *Deschampsia maderensis* (Hack. & Bornm.) Buschm. from Spain (PP = 0.99, BS = 93, data from Genbank) (Figure 1).

*Holcus lanatus* L. form a separate, moderately supported subclade (PP = 0.85, BS = 90) in the clade of *Holcus*, to which *H. mollis* L. occupies a sister position.

Construction of hybrid networks revealed a number of major ribotypes (i.e., ribotypes with more than 1000 reads) common to the studied species. Table 2 summarizes the major ribotypes of studied species.

**Figure 5:**
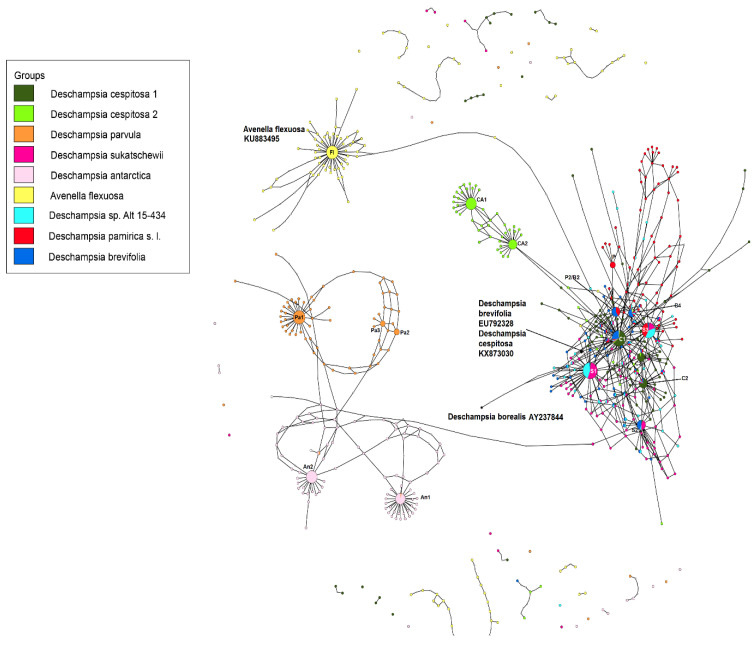

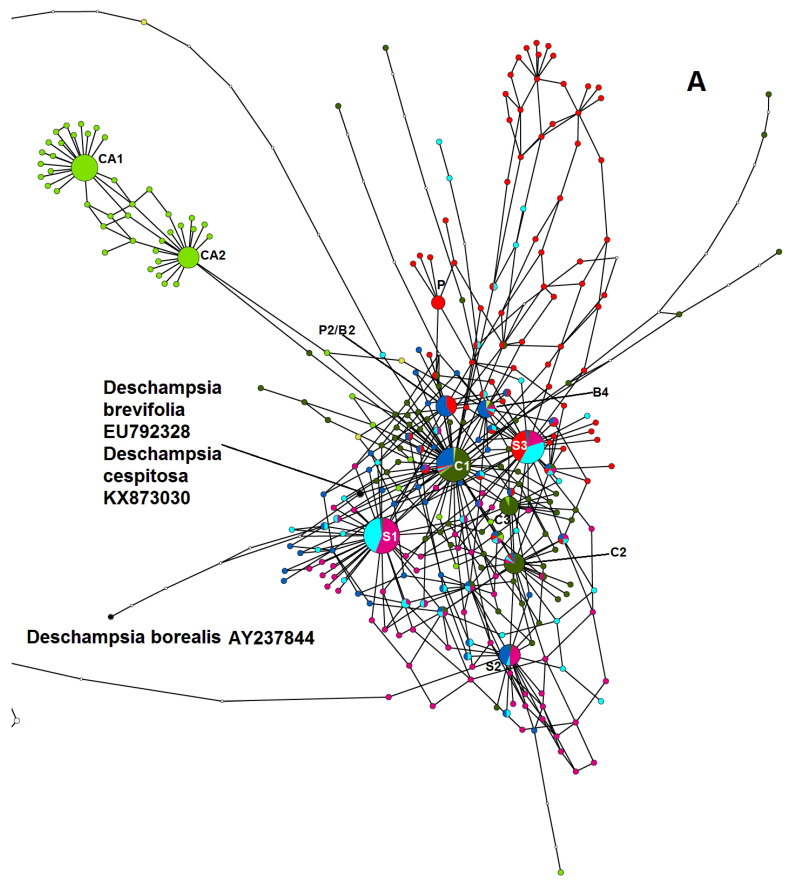

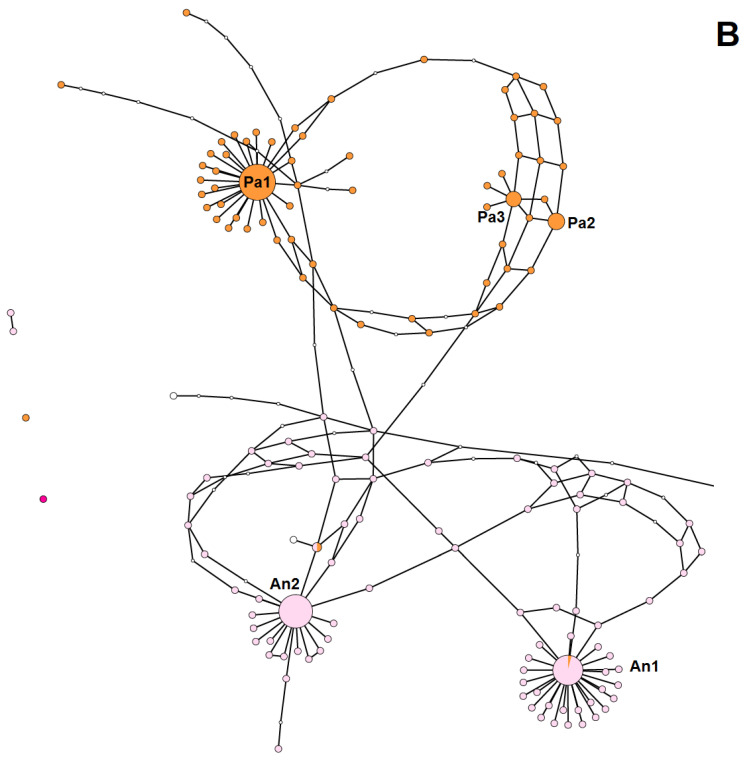
Ribotype network of the studied *Deschampsia* species. The radius of the circles on the ribotype network is proportional to the percent number of reads for each ribotype listed in Table 2. Major ribotypes (more than 1000 reads per rDNA pool) are larger than others and marked with numbers. The smaller circles correspond to ITS1 variants that have been read fewer than 1000 times. (**A**) A more detailed picture of the relationships between *Deschampsia cespitosa* and allied species. (**B**) A more detailed picture of the relationships between subantarctic species. The position of the sequences obtained from the GenBank database is shown on the picture separately.

We call the main ribotype with the largest number of reads per rDNA pool for a species, major ribotypes with more than 1000 reads per rDNA pool, and minor ribotypes with read counts less than 1000. Our data show three large subgroups within this hybrid network: *Deschampsia cespitosa* and related species, subantarctic *D. antarctica + D. parvula*, *Avenella flexuosa* (Figure 5). The main ribotype of *D. cespitosa* (sample from Great Britain) C1 (5451 reads, 24% of the total number of reads of the rDNA region) is common with the third most represented ribotype of *D. cespitosa* from Alaska (1309 reads, 7%) and with the main ribotype of *D. brevifolia* (1804 reads, 13%) (Figure 5A). Also identical to this ribotype are the minor ribotypes of *D. sukatschewii, D. pamirica*, the Altai sample *Deschampsia* sp. Alt 15-434 and *Avenella flexuosa*. The second most represented ribotype of *D. cespitosa* C2 (sample from Great Britain, 3032 reads, 13%) is homologous only to the minor ribotypes of *D. brevifolia, D. sukatschewii, D. pamirica* and sample *Deschampsia* sp. Alt 15-434, and the third ribotype C3 (2926 reads, 13%)—to the minor ribotype of *D. cespitosa* from Alaska. The main (CA1, 4988 reads, 28%) and the second major ribotypes (CA2, 3259 reads 18%) of *D. cespitosa* from Alaska are specific. At the same time, the main ribotypes of *D. sukatschewii* (3511 reads, 17%) and of the Altai sample *Deschampsia* sp. Alt 15-434 (2237 reads, 22%) are identical; they can be called S1. This ribotype is also common to the minor ribotypes of *D. cespitosa* (sample from Great Britain) and *D. brevifolia*. The second of the major ribotypes of *D. sukatschewii*, S2 (1594 reads, 8%) is common to the third major ribotype of *D. brevifolia* (1162 reads, 7%), as well as the minor ribotypes of the British sample *D. cespitosa* and the Altai sample *Deschampsia* Alt 15-434. The third major ribotype of *D. sukatschewii* (1582 reads, 8%), S3, is homologous to the second major ribotype of *Deschampsia* sp. Alt 15-434 (1731 reads, 17%), the main ribotype of *D. pamirica* s. l. (2821 reads, 18%) and minor ribotypes of *D. brevifolia* and *D. cespitosa* (sample from Great Britain). The second major ribotype of *D. pamirica* (1175 reads, 7%) is identical to the second major ribotype of *D. brevifolia* (1326 reads, 10%). We call it P2/B2. The fourth of the major ribotypes of *D. brevifolia* (1016 reads, 7%), B4, is shared with the minor ribotypes of *D. pamirica, D. sukatschewii*, British *D. cespitosa* and *Avenella flexuosa*. The third major ribotype of *D. pamirica* is specific (P, 1154 reads, 7%). In this case, the minor ribotypes of *D. pamirica* form their own subgroup within the network of *Deschampsia cespitosa* and related species. The marker sequence of *D. borealis* from the GenBank database is not identical with the ribotypes of *D. brevifolia* and mountain sample *Deschampsia* sp. Alt 15-434 from Altai Republic obtained by the NGS method. In addition, the GenBank sequences of *D. cespitosa* and *D. brevifolia* are not identical with any of the ribotypes of *D. cespitosa* and *D. brevifolia* obtained via NGS (Figure 5A).

Subantarctic representatives of *Deschampsia*, *D. antarctica* and *D. parvula* form their own network, quite far removed from *D. cespitosa* and related taxa (Figure 5B). The major ribotypes of *D. parvula* are specific, while the major ribotype of *D. antarctica* is identical to one minor ribotype of *D. parvula*.

The major ribotype of *Avenella flexuosa* forms its own “subnetwork” of ribotypes, distantly related to the network of *Deschampsia cespitosa* and related species. However, several minor ribotypes of *A. flexuosa* are part of the major ribotypes of *Deschampsia* or are found within the *Deschampsia cespitosa* network.

**Figure 6:**
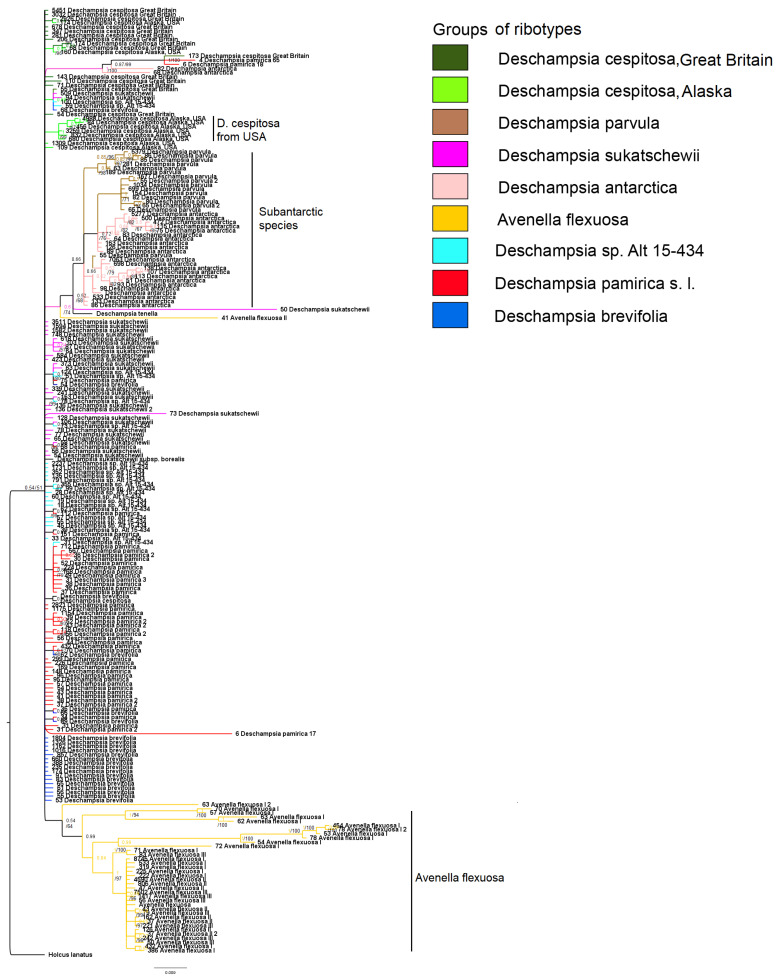
Phylogenetic tree of ribotypes of the studied *Deschampsia* and *Avenella* species obtained via NGS. Numbers before the names of species indicate the number of reads of every studied ribotype. The first index on the branch is the posterior probability in Bayesian inference, the second is the bootstrap index obtained by Maximum Likelihood algorithm. When only one index is shown on the branch, it is the posterior probability.

The tree of the ribotypes obtained via NGS demonstrates heterogeneity of the rDNA for the studied *Deschampsia* and *Avenella* samples (Figure 6).

The major ribotypes of *D. cespitosa* from Great Britain occupy an uncertain position in the low-supported clade (PP = 0.54, BS = 61) that contains all studied *Deschampsia* and *Avenella* samples. However, major ribotypes of *D. cespitosa* from the USA (Alaska) form a separate strongly supported subclade within this clade (PP = 1, BS = 99). There is also a subclade that comprises two minor ribotypes of *D. antarctica*, two minor ribotypes of *D. pamirica*, and one minor ribotype of *D. cespitosa* from Great Britain (PP = 1, BS = 100). Most of the ribotypes of the studied subantarctic species, *D. antarctica* and *D. parvula*, form a low-supported clade (PP = 0.66) in which *D. parvula* ribotypes form three (PP = 0.86, BS = 98; PP = 1, BS = 71, and PP = 0.66) subclades, two of which contain major ribotypes. One of these subclades containing the second and the third major ribotypes (PP = 1, BS = 71) is a sister to the subclade that contains the main ribotype of *D. antarctica* (PP = 0.72, BS = 76). The third subclade is a low-supported subclade (PP = 0.66) and comprises only one minor ribotype of *D. parvula* with ribotypes of *D. antarctica* including the main ribotype of *D. antarctica*. The clade of *D. antarctica + D. parvula* ribotypes groups with one minor ribotype of *D. sukatschewii* (sample from Altai Republic) and *D. tenella* sequence from GenBank (PP = 0.52, BS = 68), in turn, with the minor ribotype of *Avenella flexuosa* from Karachay Cherkessia (Caucasus, Russia) as a sister (PP = 0.6, BS = 74). The major ribotypes of mountain *Deschampsia* samples *D. pamirica*, *D. brevifolia*, and *Deschampsia* sp. Alt 15-434 occupy uncertain positions in the *Deschampsia* clade. Nevertheless, some minor ribotypes of *D. sukatschewii*, *Deschampsia* sp. Alt 15-434, *D. brevifolia*, and *D. pamirica* form specific subclades. For example, one subclade consists of the ribotypes of all these species (PP = 0.72, BS = 80). Two subclades of minor ribotypes of *D. pamirica* are well supported (PP = 0.92, BS = 93; PP = 0.94, BS = 99). Additionally, there is one low-supported clade of minor ribotypes of *D. sukatschewii* (PP = 0.64, BS = 57), one well-supported subclade of *D. pamirica* and *Deschampsia* sp. Alt 15-434 (PP = 0.84, BS = 79), and three subclades of *D. pamirica + D. brevifolia*. Most of studied samples of *Avenella flexuosa* (except for one minor ribotype) is grouped within one low-supported clade (PP = 0.54, BS = 64). Major ribotypes of *A. flexuosa* from Great Britain and Russia (Leningrad Oblast and Karachay-Cherkessia Republic) do not differ significantly from each other and fall within one strongly supported subclade (PP = 0.99, BS = 96).

**Figure 7:**
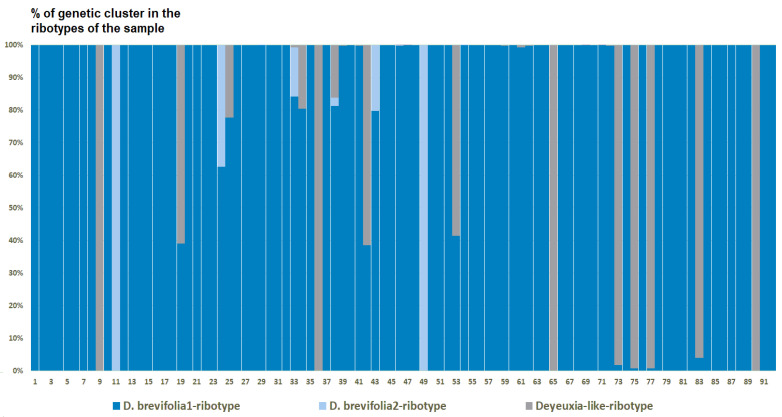
Genetic clustering of *Deschampsia cespitosa*, sample from Great Britain (K = 3). The genetic clusters correspond to the probable ancestral ribotypes within the studied sample. They are named after the most similar sequences from the GenBank database. The number of columns corresponds to the number of ribotypes within the sample.

Analysis of the ribotype structure of the studied species of *Deschampsia* revealed rather significant differences and suggested possible ancestral taxa. Our estimated number of genetic clusters reflects ribotype composition within the appropriate sample and not between samples. The genetic clusters (estimated ancestral ribotypes) are named after the species to which they were compared via BLAST (https://blast.ncbi.nlm.nih.gov/Blast.cgi, accessed on 20 May 2024). The optimal quantity of genetic clusters (estimated ancestral ribotypes) (K) computed based on SNP between ribotypes is also different in the samples of the studied species. The ribotype pool of *D. cespitosa* from Great Britain (K = 3) is composed of two estimated ancestral ribotypes corresponding to *D. brevifolia*-ribotype variants and one *Deyeuxia*-like-ribotype variant that is similar to various species of *Deyeuxia* and *Calamagrostis* Adans., e. g., *Deyeuxia aucklandica* (Hook.f.) Zotov and *Calamagrostis nivicola* (Hook.f.) Hand.-Mazz (Figure 7).

**Figure 8:**
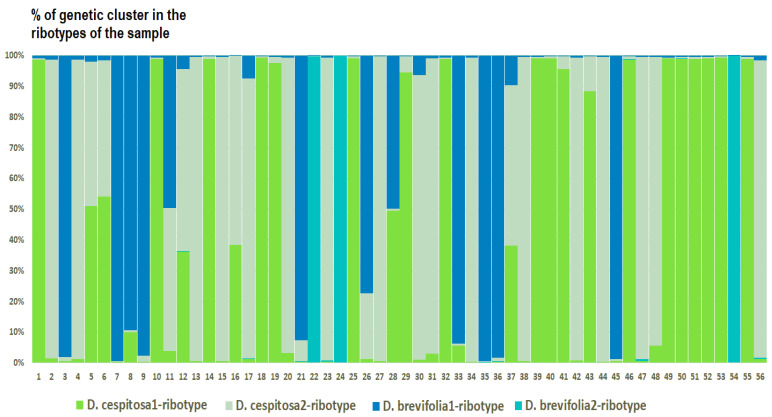
Genetic clustering of *Deschampsia cespitosa*, sample from Alaska, USA (K = 4). The genetic clusters correspond to the probable ancestral ribotypes within the studied sample. They are named after the most similar sequences from the GenBank database. The number of columns corresponds to the number of ribotypes within the sample.

**Figure 9:**
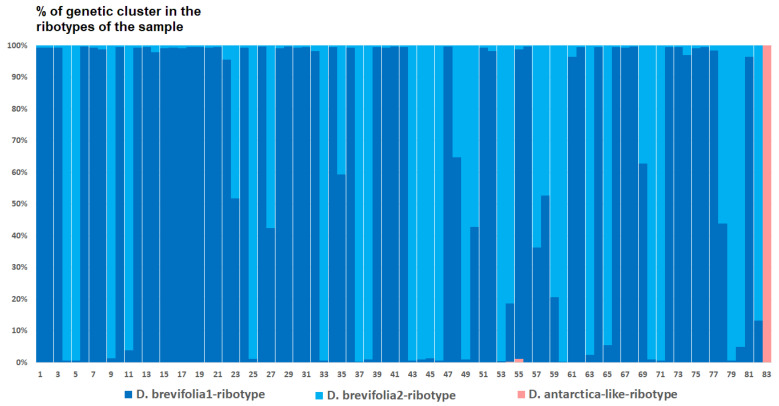
Genetic clustering of *Deschampsia pamirica* s. l. from Altai Republic, Russia (K = 3). The genetic clusters correspond to the probable ancestral ribotypes within the studied sample. They are named after the most similar sequences from the GenBank database. The number of columns corresponds to the number of ribotypes within the sample.

**Figure 10:**
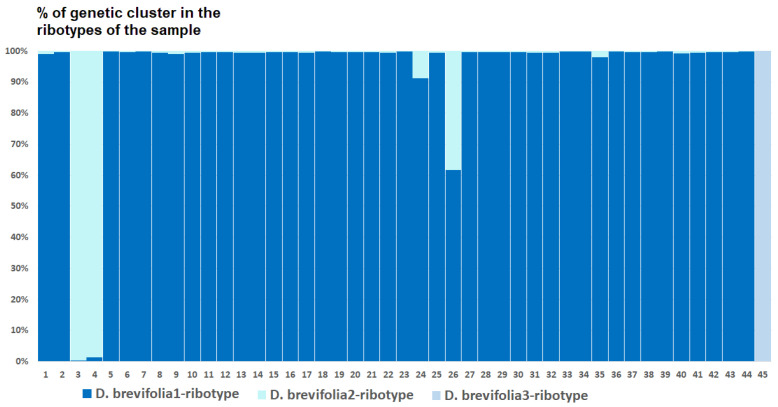
Genetic clustering of *Deschampsia* sp. Alt 15-434, sample from Altai Republic, Russia (K = 3). The genetic clusters correspond to the probable ancestral ribotypes within the studied sample. They are named after the most similar sequences from the GenBank database. The number of columns corresponds to the number of ribotypes within the sample.

**Figure 11:**
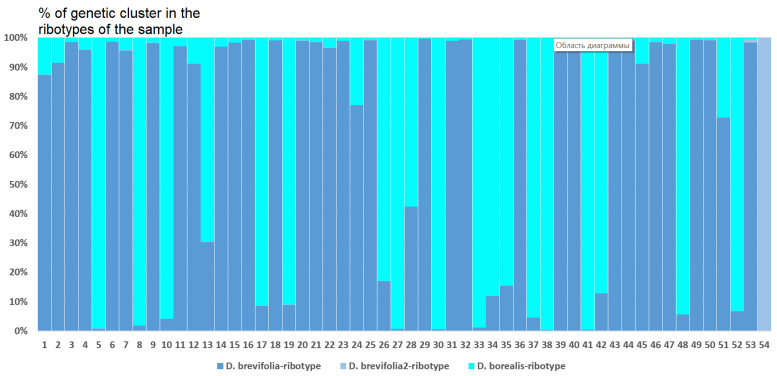
Genetic clustering of *Deschampsia brevifolia* (K = 3). The genetic clusters correspond to the probable ancestral ribotypes within the studied sample. They are named after the most similar sequences from the GenBank database. The number of the columns corresponds to the number of ribotypes within the sample.

**Figure 12:**
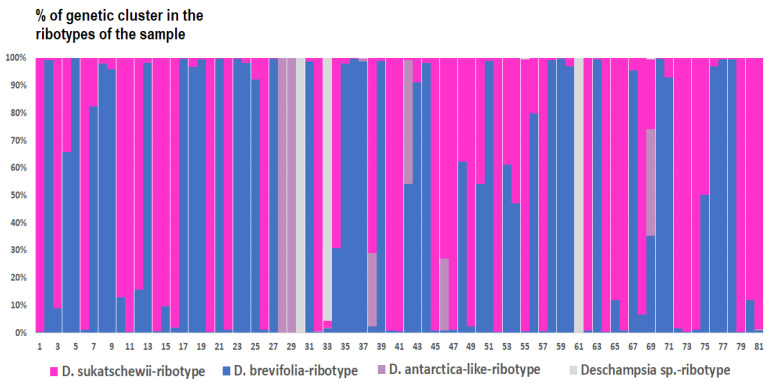
Genetic clustering of *Deschampsia sukatschewii* s. l. (sample from Altai Republic, Russia) (K = 4). The genetic clusters correspond to the probable ancestral ribotypes within the studied sample. They are named after the most similar sequences from the GenBank database. The number of the columns corresponds to the number of ribotypes within the sample.

**Figure 13:**
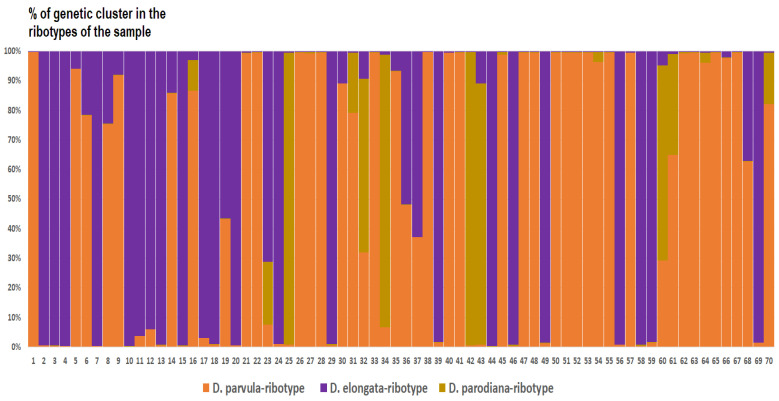
Genetic clustering of *Deschampsia parvula* (K = 3). The genetic clusters correspond to the probable ancestral ribotypes within the studied sample. They are named after the most similar sequences from the GenBank database. The number of the columns corresponds to the number of ribotypes within the sample.

**Figure 14:**
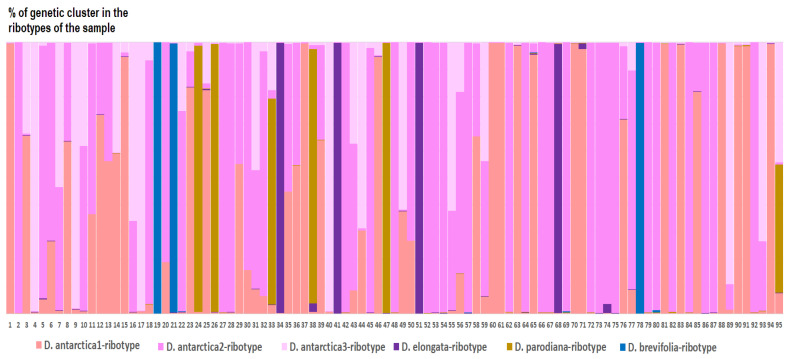
Genetic clustering of *Deschampsia antarctica* (K = 6). The genetic clusters correspond to the probable ancestral ribotypes within the studied sample. They are named after the most similar sequences from the GenBank database. The number of columns corresponds to the number of ribotypes within the sample.

**Figure 15:**
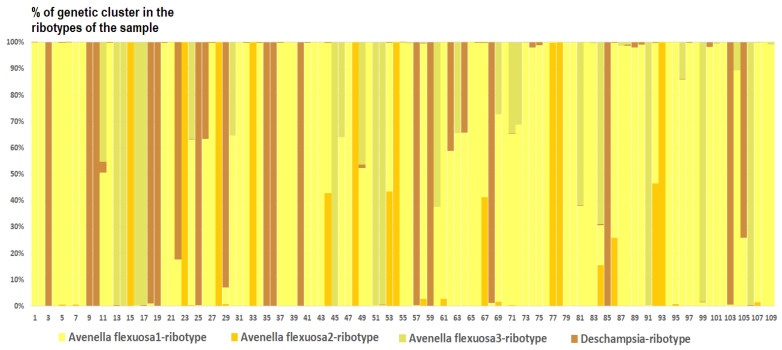
Genetic clustering of *Avenella flexuosa*, sample from Great Britain (K = 4). The genetic clusters correspond to the probable ancestral ribotypes within the studied sample. They are named after the most similar sequences from the GenBank database. The number of columns corresponds to the number of ribotypes within the sample.

**Figure 16:**
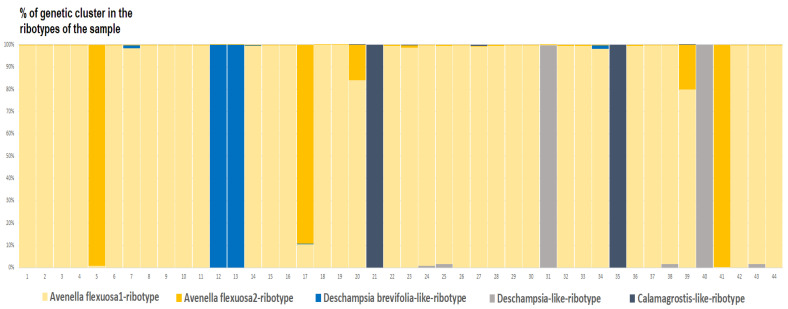
Genetic clustering of *Avenella flexuosa*, sample from Leningrad Oblast, Russia (K = 5). The genetic clusters correspond to the probable ancestral ribotypes within the studied sample. They are named after the most similar sequences from the Genbank database. The number of columns corresponds to the number of ribotypes within the sample.

**Figure 17:**
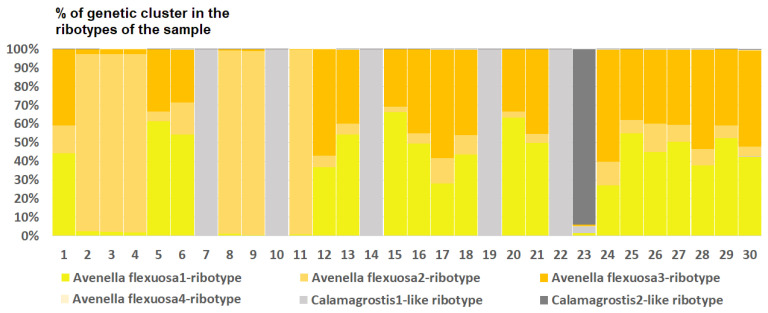
Genetic clustering of *Avenella flexuosa*, sample from Karachay-Cherkessia Republic, Russia (K = 6). The genetic clusters correspond to the probable ancestral ribotypes within the studied sample. They are named after the most similar sequences from the GenBank database. The number of columns corresponds to the number of ribotypes within the sample.

*Deschampsia cespitosa* from Alaska, USA (K = 4), on the contrary, has two *D. cespitosa*- and two *D. brevifolia*-like ancestral ribotypes (Figure 8). Altaian samples of *D. pamirica* s. l. (K = 3) and *Deschampsia* sp. Alt 15-434 (K = 2) consist of two different ribotype variants more or less corresponding to *D. brevifolia* s. l. (Figure 9 and Figure 10), but in *D. pamirica*, there is also one estimated ancestral ribotype that is similar to *D. antarctica* (Figure 9). *Deschampsia brevifolia* s. l. has K = 3; two *D. brevifolia*-like-estimated ancestral ribotypes and one estimated ribotype that corresponds to *D. borealis* (Figure 11). *Deschampsia sukatschewii* s. l. from Altai Republic is characterized by K = 4: *D. sukatschewii* and *D. brevifolia*-ribotypes along with *D. antarctica*-like ribotype and *Deschampsia*-ribotype that is rather distant to either *D. cespitosa* or *D. brevifolia* ribotypes (Figure 12). The studied sample of *D. parvula* (K = 3) consists of *D. parvula-*, *D. elongata-*, and *D. parodiana*-ribotypes (Figure 13). The most diverse is the studied sample of *D. antarctica* (K = 6). It has two variants of *D. antarctica* ribotypes, *D. parodiana* and *D. elongata* ribotypes, and, surprisingly, *D. brevifolia*-like ribotypes (Figure 14). *Avenella flexuosa* samples from different regions have diverse ribotype composition according to the genetic clustering computed in program Structure 2.3. Sample of *Avenella flexuosa* from Great Britain (K = 4) consists of three estimated ancestral ribotypes corresponding to *A. flexuosa* but also of one *Deschampsia* ribotype (Figure 15). *Avenella flexuosa* from Russia, Leningrad Oblast (K = 5) has two estimated *A. flexuosa* ribotypes, two *Deschampsia* ribotypes (one of them is more closely to *D. brevifolia*), and one *Calamagrostis*-like ribotype (Figure 16). *Avenella flexuosa* sample from Karachay-Cherkessia Republic demonstrates six estimated ancestral ribotypes, K = 6. Four of them correspond to A. *flexuosa* and two correspond to *Calamagrostis*-related ribotypes (Figure 17).

## 3. Discussion

Our subject of study, *Deschampsia*, is a circumpolar genus that includes species connected by numerous morphological transitions [8,12,13]. Most probably, the process of speciation by hybridization in this genus has not yet been completed. The diploid number of chromosomes in the *Deschampsia* species is 26, which is quite unusual for grasses (such a number of chromosomes, uncharacteristic for Poaceae, was also found in representatives of the genera *Rostraria* Trin., *Hainardia* Greuter and *Nardus* L. [1]). The genus *Rostraria* belongs to the subtribe Koeleriinae Asch. et Graebn. tribes Poeae s. l., which is in the stage of active hybridization. The genera *Hainardia* and *Nardus* are evolutionarily isolated, more or less morphologically primitive, with *Nardus* belonging to the tribe Nardeae W.D.J.Koch, which is at the base of the Pooideae family lineage [4]. We see that all known genera of grasses with 2n = 26 are complex hybrids located at the boundaries of their tribes.

Such a karyotype in the genus *Deschampsia* could be considered a manifestation of dysploidy resulting from the loss or fusion of a pair of chromosomes in the original 2n = 28 tetraploid genome [17]. This may indicate an allopolyploid origin of the genus as a whole, since two different ancestral taxa with different karyotypes participated in the formation of the genus, resulting in the formation of a heteromorphic chromosomal complex [17,20,21,22]. Modern hybridogenous species of *Deschampsia* have 2n = 52 and the karyotype of species of this genus is quite conservative [16,17,18,20,21]. At the same time, the tetraploid chromosome number 2n = 52 can be observed both in species of the genus from high latitudes, such as *D. brevifolia* [23], and in the type species of the genus *D. cespitosa* [9]. Hybridization processes occurring in the genus *Deschampsia* and the associated concerted evolution of rDNA [24,25] may make it difficult to accurately construct phylogenetic schemes.

Our results of molecular phylogenetic analysis of the ITS1–5.8S rRNA gene–ITS2 region obtained by the Sanger method indicate three large evolutionary lines in the genus (Figure 1). All studied northern and Siberian representatives of the genus belong to the subclade *D. cespitosa* s. l. The lack of resolution within the subclade probably indicates significant hybridization processes occurring among most species of tussock grass in the Northern Hemisphere. One of our samples of *Deschampsia sukatschewii* from Yakutia occupies an uncertain position in the genus according to our data, which is associated with a number of substitutions in the primary sequence of the ITS1–5.8S rRNA gene–ITS2 region (Figure 1). This may indicate the hybrid status of *D. sukatschewii*. As a result, in the Yakut sample of *D. sukatschewii*, a sequence from some Arctic species was amplified during direct sequencing, whereas the Altai sample contains more sequence variants related to *D. cespitosa* s. str.

In the “southern *Deschampsia*” subclade, internal resolution is higher (Figure 1), possibly indicating a factor of the geographic isolation of species in the subantarctic region. In addition, some researchers claim that hybrid subantarctic tussock grasses with 2n = 52 could have been formed independently several times [17]. Different subclades of the subantarctic species reflect recent differentiation processes and local hybridization events [16,17,18]. Moreover, diverse subantarctic species (e.g., *D. antarctica* from Falkland Islands, Antarctic Peninsula, and *D. chapmanii* from New Zealand) could migrate from different locations (from South America and Southeastern Asia, respectively). Note that along with the subantarctic complex *D. antarctica + D. parvula*, there was also *D. danthonioides*, a specimen from Washington State, USA. Such a relationship may tell us about the colonization of the Southern Hemisphere by grass species along the Cordillera chain, since *D. danthonioides* is a widespread species on the west coast of America (see also [26]). In the second branch of southern *Deschampsia*, we see two samples of *D. setacea* from Norway (sect. *Aristavena* (F.Albers & Butzin) Tzvelev, data from GenBank). This is another case of interpolar disjunction among grass species—the close relationship of Arctic and subantarctic species (a phenomenon that we have already described earlier in the study of North Pacific and subantarctic species of bluegrass [27]). This phenomenon cannot yet be precisely explained, but it has been found in a fairly large number of plant species. We consider an important and interesting fact that *D. setacea* has an unusual chromosome number for the genus, 2n = 14 [28]. This most likely indicates the closeness of *D. setacea* to the ancestral taxa of the entire genus *Deschampsia*. According to morphological criteria, *D. setacea* has a geniculate awn on the lemma callus, which is probably the original character in the tribe Poeae s. l. (Aveneae s. str. and Poeae s. str.).

The evolution of boreal and subarctic species of the genus *Deschampsia* seems quite complex and interesting. According to morphological and geographical criteria, *D. cespitosa* s. str. and *D. sukatschewii* (=*D. cespitosa* subsp. *orientalis* Hultén) arose in parallel during the Alpine orogeny [13]. *Deschampsia cespitosa* originated in the western part of Eurasia, and *D. sukatschewii* in the eastern part [13]. Later, *D. sukatschewii* probably moved along the northern coasts of Eurasia far to the west and formed secondary hybrids with *D. cespitosa*: *D. glauca* and *D. borealis* [13]. When analyzing ITS sequences using the Sanger method, precisely because of numerous cases of hybridization, we did not identify any serious differences between northern (subarctic and Arctic) *Deschampsia* species and those of the temperate zone. However, our next-generation sequencing (NGS) data revealed important differences in the ribotype composition of *D. cespitosa* s. str., *D. sukatschewii*, and northern and Altai species (Figure 5A and Figure 6). *Deschampsia cespitosa* (specimen from Great Britain) forms a network of major ribotypes (the number of reads is more than 1000), and its main ribotype is also the main one in the high-arctic *D. brevifolia* (Figure 5A). At the same time, *D. cespitosa* from Alaska has specific major ribotypes not common with any other ribotype. According to genetic cluster analysis that reveals possible ancestral ribotypes, *D. cespitosa* from Alaska has *D. cespitosa* and *D. brevifolia* ribotypes, whereas *D. cespitosa* from Great Britain has only *D. brevifolia*-ribotype variants from the *Deschampsia*-like ribotype pool (Figure 7 and Figure 8). Here, it is useful to mention the assumption of R.V. Kamelin about the existence of plant introgressive–interspecific complexes in nature [29]. Most probably, *D. cespitosa* from Alaska was growing in the zone of introgressive hybridization. The sample from Great Britain could in fact be the introgressive hybrid morphologically intermediate between *Deschampsia cespitosa* s. str. and *D. brevifolia* with rDNA that retained only from the latter species. Our phylogenetic pattern among different samples of *D. cespitosa* and allied species probably reflects a different evolutionary history of the geographical lines within a large complex of *D. cespitosa* s. l., as was shown according to plastome data [14].

*D. sukatschewii* (the sample from Altai Republic) retained its own major ribotypes in the genome set, most likely indicating a separate origin of its ancestral taxa from *D. cespitosa* (Figure 5A). Later, introgressive hybridization apparently occurred with *D. cespitosa* s. l., but quite a long time ago, since there are very few ribotypes of *D. cespitosa* that are common with the major ribotypes of *D. sukatschewii*. These data support a hypothesis by Tzvelev [8,13] based on morphological criteria. It is likely that geographically more distant samples of *D. sukatschewii* may have even fewer rDNA ribotypes from *D. cespitosa* s. str. It is also interesting that the main ribotype of *D. sukatschewii* is identical to the main ribotype of that very isolated in the morphological characteristics specimen *Deschampsia* Alt 15-434, which was collected on the bank of the river Yustyt in the Kosh-Agach region of the Altai Republic. This specimen is well distinguished by the large size of the entire plant, very long glumes, and a long but compressed panicle, somewhat reminiscent of the Eurasian hypoarctic species *D. obensis* Roshev. Perhaps the sample *Deschampsia* sp. Alt 15-434 is a high-mountain Altai hybrid involving *D. sukatschewii* s. l. The second putative parent taxon may be the Altai *D. pamirica* s. l., differing from *D. koelerioides* by an oblong and elongated panicle. In Altai, this Central Asian species is located at the northwestern border of its range. Perhaps this circumstance led to more intense processes of allopolyploidization and hybridization in the Pamir *Deschampsia* taxa, and the complex of ribotypes that differs from that of the rest may be of Central Asian origin. We also suppose the probable hybridization of the Altai *D. pamirica* with a certain ancestral taxon related to *D. brevifolia* (Figure 5).

High Arctic *D. brevifolia* is related to *D. glauca* and *D. obensis* by morphological transitions [8] and could have originated from secondary hybridization of species related to *D. cespitosa* s. str. and *D. sukatschewii* that could migrate to the north [13]. We need to note that the sequences of *D. brevifolia* and its related species, *D. borealis*, are rather different from each other because of the sample location. Because of this, the sequences from GenBank obtained by the Sanger method are not identical with those obtained via NGS (Figure 5A). Comparing the genetic clusters (probable ancestral ribotypes) obtained via program Structure 2.3, we found that the ribotypes of all mountain samples of *Deschampsia* more or less correspond to the ITS sequences of *D. brevifolia* from the GenBank database showing that *D. brevifolia* or some extinct but related taxon could take place in the formation of most parts of the Altaian species (Figure 9 and Figure 12). In addition, *D. pamirica* could hybridize in some distant past with some species that were related to Antarctic *Deschampsia*, as the genetic clustering has shown (Figure 9). The genetic structure of the studied sample of *D. brevifolia* s. str. contains some ribotypes that correspond to *D. borealis* (data from Genbank) (Figure 11). Thus, Arctic species *D. brevifolia* can form an introgressive complex with *D. borealis* that has been confirmed by the occurrence of transitional populations [8]. Genetic clustering of the studied sample of *D. sukatschewii* from Altai Republic demonstrates the presence of both *D. sukatschewii* and *D. brevifolia* ribotypes (Figure 12). It points to the fact that *D. sukatschewii* nevertheless differs from *D. brevifolia* by rDNA and also that modern Siberian *Deschampsia* species went through successive rounds of hybridization with northern ones.

The genetic clustering revealed some clusters that are close to *D. antarctica* as well. This can reflect the ancient hybridization with taxa that afterwards gave rise to the Antarctic *Deschampsia* complex of species.

The Antarctic species *D. antarctica* and *D. parvula*, according to the results of NGS analysis, are rather distantly related to the *Deschampsia* species of the Northern Hemisphere, and are also well separated from each other (Figure 5B). Moreover, each species in its set contains two families of ribotypes that are quite different from each other. Judging by the structure of the ribotypes, if hybridization occurred in their evolutionary history, it happened a long time ago. Also, the minor ribotypes of *D. parvula*, common with *D. antarctica*, could have been inherited from common ancestors. Most likely, Antarctic and subantarctic species developed independently of the northern species of the *D. cespitosa* s. l. complex quite a long time ago. Nevertheless, some ribotypes in the rDNA pool of *D. antarctica* could be related to those of arctic *D. brevifolia* s. l. although significantly changed (Figure 14). They can be inherited from some species that was a descendant of arctic *Deschampsia* in the distant past. We need to note that polyploid *D. antarctica* and *D. parvula* samples have in their rDNA pool *D. elongata*- and *D. parodiana*-like ribotypes (data obtained from genetic clustering, Figure 14 and Figure 15). Two latter species are South American; *D. parodiana* was previously treated as species of *Calamagrostis*. This fact further supports the hypothesis of the South American origin of Antarctic *Deschampsia* species.

In addition to *Deschampsia* species, we included in the analysis *Avenella flexuosa* (formerly *Deschampsia flexuosa* (L.) Trin.), a species from a closely related genus. Until recently, the question of placing *Avenella* into a separate genus remained unresolved in taxonomy. It should be noted that *A. flexuosa* has a different chromosome number from species of the genus *Deschampsia*, 2n = 28 [30], and the karyotypic structure is very different from the karyotypes of *Deschampsia* species [31]. Our molecular phylogenetic studies confirm earlier data leading to the treatment of *A. flexuosa* as a species from a separate genus [32,33]. It is likely that the genus *Avenella* is close to the ancestor of the hybridogenous genus *Deschampsia*, especially considering the number of chromosomes. However, quite unexpectedly, among the pool of ITS1 sequences in the sample of *A. flexuosa* (Great Britain), minor components (54 reads) related to ribotypes of *Deschampsia cespitosa* were discovered (Figure 5A and Figure 6). This may suggest that genetic barriers to intergeneric hybridization are probably not so strict here. Recently, we have received some evidence of possible intergeneric introgressive hybridization in the tribe Poeae s. l.: this is the presumed origin of *Phippsia concinna* (Th.Fr.) Lindeb. from a species related to *Coleanthus subtilis* Seidl ex Roem. & Schult. and one of the Arctic representatives of the genus *Puccinellia* Parl. [34], and possible introgressive absorption of the genome of *Poa diaphora* Trin. s. l. (=*Eremopoa persica* (Trin.) Roshev.) by a certain species of the genus *Zingeria* P.A. Smirn. with the formation of *Zingeria trichopoda* (Boiss.) P.A. Smirn. [35]. Taking into account that the difference between *Poa diaphora* and the genus *Zingeria*, according to molecular phylogenetic data is at the level of two subtribes [4,5], intergeneric hybridization between very close genera within a single subtribe Airinae is quite likely. At the same time, a very small number of *Deschampsia* sequences in the genome set of *Avenella flexuosa* may indicate that hybridization took place in the very distant past, perhaps even before the separation of these genera. Another possible hybridization event demonstrates the position of *Deschampsia* (=*Avenella*) *foliosa* (Figure 1). This species is endemic to Azorean Isles and was at first described by Hackel [36] as a member of the genus *Deschampsia.* Then it was considered as close to *Avenella flexuosa* because of the leaf and inflorescence features and was transferred to the genus *Avenella* [37]. Nevertheless, ITS data from GenBank show that *D. foliosa* is a relative to *D. cespitosa* (Figure 1). Thus, *Deschampsia foliosa* can in fact be the intergeneric hybrid originated from introgression. This is corroborated by the position of *D. maderensis* which is closely related to *D. foliosa* but falls within *Avenella* clade according to ITS data (Figure 1).

From the genetic clustering analysis, we probably see a more ancient hybridization pattern with some very interesting points. The most important thing is that one sample of *D. cespitosa* (from Great Britain) and two samples of *Avenella flexuosa* (from Leningrad Oblast and Karachay-Cherkessia Republic, Russia) have possible tracks of intercrossing with some *Deyeuxia* and *Calamagrostis* species (Figure 15, Figure 16 and Figure 17). We need to note that *Deyeuxia*/*Calamagrostis*-like ribotypes present in rDNA of *D. cespitosa* from Great Britain as well these presenting in rDNA of *A. flexuosa* are rather changed due to the post-hybridization processes [38] and are not identical to ITS sequences of the modern *Deyeuxia* species. Previous phylogenetic works did not recognize *Deschampsia* and *Avenella* as the part of introgression complex with the genera of *Aveninae* or *Koeleriinae*, they rather treated *Deschampsia* and *Avenella* (*Deschampsia* s. l.) as the part of hybrid subtribe Aristaveninae that originated from some intercrossing between some Aveneae and Festuceae Dumort. taxa without naming precise ancestors of *Deschampsia* s. l. [6]. Studies on chromosome mapping revealed some American *Deyeuxia* species, for example, *D. eminens* J.Presl as very close relatives and possible ancestors of the genus *Deschampsia* [17]. This was confirmed by sequence analysis as well [18] but *D. eminens* does not bear resemblance to *Deyeuxia* s. str. lineage that is close to *Calamagrostis* [39]. We can assume that some taxa of the genus *Deyeuxia* can also be of hybrid origin and *Deschampsia* can be the genus with *Deyeuxia* or some *Calamagrostis* species as an ancestor from the line of tribe Aveneae Dumort. s. str. The subtribe Airinae to which *Deschampsia* and *Avenella* belong was previously placed near subtribe Koeleriinae that is related to Aveninae and related subtribes [1,3]. Probably the hybridization between *Deschampsia*, *Avenella*, and some *Calamagrostis*/*Deyeuxia* species took place long ago.

## 4. Materials and Methods

### 4.1. DNA Sampling for Analysis

For a molecular phylogenetic study, we obtained 12 species of *Deschampsia* and 1 species of *Avenella*. The samples were collected during expeditions of the Laboratory of Biosystematics and Cytology of the Komarov Botanical Institute, and also received via the Engelhardt Institute of Molecular Biology, Moscow. Information about the collected samples and sequence reads is presented in Table 1. Additionally, for phylogenetic analysis of sequences obtained by the Sanger method, sequences of 11 more *Deschampsia* species were obtained from the GenBank database.

It should be noted that of the 23 species of *Deschampsia* that we used for analysis, 8 species, in some interpretations of certain authors, were considered subspecies of the species *D. cespitosa* s. l. Of these, *D. koelerioides* Regel is now considered an accepted species. The other seven are *D. alpina*, *D. borealis*, *D. brevifolia*, *D. glauca* Hartm., *D. koelerioides*, *D. pamirica* Roshev., *D. sukatschewii* (Popl.) Roshev., and *D. submutica* (Trautv.) O.D.Nikif. (https://powo.science.kew.org/, accessed on 12 November 2023).

### 4.2. DNA Isolation and Sequencing by the Sanger Method

Genomic DNA isolation and sequencing of the ITS1–5.8S rDNA–ITS2 region by Sanger method was carried out at the center for the collective use of scientific equipment “Cellular and molecular technologies for the study of plants and fungi” of the Komarov Botanical Institute, St. Petersburg. Plant genomic DNA was isolated with the aid of the Qiagen Plant Mini Kit (Qiagen Inc., Hilden, Germany) according to the user manual. The polymerase chain reaction was carried out with primers ITS 1P [40] and ITS 4 [41] with the following parameters: initial denaturation 95 °C for 1 min, then 35 cycles: 95 °C in for 30 s, 55–56 °C for 30 s, 72 °C for 30 s, and final elongation for 5 min. Sequencing was carried out on ABI PRIZM 3100 equipment using the BigDyeTM Terminator Kit ver. 3.1 (168 Third Avenue, Waltham, MA USA).

### 4.3. Molecular Phylogenetic Analysis of the Sequences Obtained by the Sanger Method

The resulting chromatograms were analyzed by Chromas Lite version 2.01 (Technelysium co.) and then the sequences were aligned by Muscle algorithm [42] included in the MEGA v. 11.0.13 software package [43]. Evolutionary models for the studied set of sequences were calculated using the jModeltest program v. 2.1.10 [44]. Indel regions were coded by SeqState 1.4.1 [45] and included in the alignment file. Bayesian analysis was performed by Mr. Bayes 3.2.2 [46], calculation parameters: GTR+I+G, 1 million iterations, the first 25% of trees were excluded as “burn-in”. Maximum likelihood analysis was performed with the aid of iqtree 2.3.6 [47] under the GTR+I+G model, fast bootstrap option, 1000 generations.

### 4.4. Next-Generation Sequencing

NGS was carried out at the Center for Shared Use “Genomic Technologies, Proteomics and Cell Biology” of the All-Russian Research Institute of Agricultural Microbiology on an Illumina Platform MiSeq. We used 15 µL of PCR mix containing 0.5–1 unit of activity of Q5^®^ High-Fidelity DNA Polymerase (NEB, Ipswich, MA, USA), 5 pM of forward and reverse primers, 10 ng of DNA template, and 2 nM of each dNTP (Life Technologies, ThermoScientific, Waltham, MA, USA). The PCR was carried out using primers ITS 1P [40] and ITS 2 [41] under the following parameters: initial denaturation 94 °C for 1 min, followed by 25 cycles of 94 °C for 30 s, 55 °C for 72 °C for 30 s, and a final elongation for 5 min. PCR products were purified using AMPureXP (Beckman Coulter, Indianapolis, IN, USA). Further preparation of the libraries was carried out in accordance with the manufacturer’s MiSeq Reagent Kit Preparation Guide (Illumina) (https://support.illumina.com/documents/documentation/chemistry_documentation/16s/16s-metagenomic-library-prep-guide-15044223-b.pdf (accessed on 12 November 2023)). The libraries were sequenced, according to the manufacturer’s instructions, on an Illumina MiSeq instrument (Illumina, San Diego, CA, USA) using a MiSeq^®^ ReagentKit v. 3 (600 cycle) with pair-end reading (2 × 300n).

### 4.5. Molecular Phylogenetic Analysis of NGS Data

The obtained pool of raw sequences was trimmed with the aid of Trimmomatic [48] included in Unipro Ugene [49] using the following parameters: PE reads; sliding window trimming with size 4 and quality threshold 12; and minimal read length 130. Then paired sequences were combined and dereplicated and sorted by vsearch 2.7.1 [50]. The resulting sequences formed ribotypes in the whole pool of genomic rDNA; they were sorted according to their frequency. For our analysis, we established a threshold of 10 reads per pool of rDNA. The sequences were aligned using MEGA v. 11.0.13 [43]; a ribotype network was built in TCS 1.2.1 [51] and visualized in TCS BU [52]. In addition, we made a phylogenetic tree of the obtained ribotypes by Bayesian and Maximum Likelihood methods using GTR+G model. Bayesian analysis was conducted with 2–5 millions of generations by Mr. Bayes 3.2.2 [46]. ML analysis was conducted using GTR+G model with the aid of iqtree 2.3.6 [47]. In this case, we set a threshold of 30 reads per rDNA pool.

As we see, each studied sample of *Deschampsia* analyzed by the NGS method has its own population of ribotypes (marker sequences). These ribotypes, in turn, can be inherited from different species and even genera forming the subgenomes in the total rDNA pool. We applied population analysis methods to the set of ribotypes within each studied sample. The resulting pattern depicts the probable origin of the ribotypes within a sample from ancestral taxa similar to that when geographic differentiation is revealed by analyzing gene sequences of different samples of a species. To analyze the ribotype pattern of each sample separately, we conducted a model-based clustering method using the program Structure 2.3 [53]. The sequence files in fasta-format were converted to the Structure input files by R script for diploid organisms (https://sites.google.com/site/thebantalab/tutorials#h.e9y185vac91q, accessed on 20 May 2024). We tested the rDNA pool of each sample obtained via NGS for revealing single-nucleotide polymorphisms (SNPs) that can be phylogenetically significant. The genetic clusters computed by Structure more or less correspond to the ribotypes of the sample and reflect probable ancestral taxa that gave origin to the ribotypes of the studied species. Each run of Structure 2.3 [53] used the following parameters: burn-in period of 10,000 replicates, 50,000 MCMC replicates after burn-in, 3 iterations of each burn-in computing, and K (number of hypothetic ancestral ribotypes) was set from 2 to 8. The correct number of K was then calculated with the aid of Evanno test [54] implemented in StructureHarvester Python Script [55]. The resulting K is a number of hypothetical ancestral ribotypes that are present in our polyploid sample; mostly they represent the major ribotypes obtained via NGS with their derivatives, but also some minor ribotypes. Results of the clustering were subsequently visualized in MS Excel 2016. The ribotype pool of each sample was analyzed separately; analyzed ribotypes within the sample are shown on the figures by columns. Number of the columns corresponds to the number of ribotypes within the sample (from 30 to 109). We compared each estimated genetic cluster with sequences from the GenBank database and named these clusters according to the sequences from GenBank that were the most similar to them.

## 5. Conclusions

Our data indicate that the evolution of the genome of the genus *Deschampsia* included polyploidy (see also [31] for cytogenetic data). We also see the presence of at least two ancestral lines within the large complex of *D. cespitosa*, eastern and European, which supports the assumption of N. N. Tzvelev [13] about the independent origin of *D. cespitosa* s. str. and *D. sukatschewii* (*D. cespitosa* subsp. *borealis*) with *D. brevifolia* as a probable derivative. Our data confirm previous findings from cytogenetic markers that the genomes of *D. sukatschewii* and *D. cespitosa* are more closely related compared to *D. antarctica* [56]. The genus *Avenella* is separated from *Deschampsia* by molecular data; nevertheless, it was involved in the hybridization processes with the species of *Deschampsia*. We see that the process of speciation in this genus is probably not yet complete. Our data obtained by modern methods allowed us to confirm the truth of a statement made more than a century ago by W. Bateson ([57], p. 91): “When formerly we looked at a series of plants produced by hybridization we perceived little but bewildering complexity. We knew well enough that behind this complexity order and system were concealed”.

## Figures and Tables

**Table 1:** Sequences analyzed in the present study and their numbers in GenBank.

Species	GenBank Number		Country of Origin
	ITS1–5.8S rRNA Gene–ITS2, Sanger Method	18S rRNA Gene–ITS1–5.8S rRNA Gene, NGS	
*Deschampsia antarctica* E.Desv.	OR900972		Great Britain, Falkland Islands
*Deschampsia antarctica* E.Desv.	OR900973		Great Britain, Falkland Islands
*Deschampsia antarctica* E.Desv.		OR908158–OR908252	Great Britain, Falkland Islands, Weddel Island
*Deschampsia baicalensis* Tzvelev	OR903200		Russia, Altai Republic
*Deschampsia brevifolia* R.Br.	OR900968	OR908382–OR908435	Russia, Krasnoyarsk Krai, Bolshevik Island
*Deschampsia cespitosa* (L.) P.Beauv.	OR900970	OR907857–OR907948	Great Britain, Wales
*Deschampsia cespitosa* (L.) P.Beauv.	OR901684		Russia, Altai Republic
*Deschampsia cespitosa* (L.) P.Beauv.		OR907949–OR908004	USA, Alaska
*Deschampsia danthonioides* Munro	OR900974		USA, Washington state
*Deschampsia glauca* Hartm.	OR903202		Russia, Tyva Republic
*Deschampsia koelerioides* Regel	OR900969		Russia, Altai Republic
*Deschampsia pamirica* Roshev	OR903201	OR908299–OR908381	Russia, Altai Republic
*Deschampsia parvula* E.Desv.	OR900975	OR908005–OR908075	Great Britain, Falkland Islands
*Deschampsia submutica* (Trautv.) O.D.Nikif.	OR903199		Russia, Altai Republic
*Deschampsia sukatschewii* (Popl.) Roshev.	OR900971	OR908076–OR908157	Russia, Altai Republic
*Deschampsia sukatschewii* (Popl.) Roshev.	OR900967		Russia, Yakutia
*Deschampsia* sp. Alt 15-434		OR908253–OR908298	Russia, Altai Republic
*Avenella flexuosa* (L.) Drejer	OR901685	PQ269223–PQ269266	Russia, Karachay-Cherkessia Republic
*Avenella flexuosa* (L.) Drejer	OR901686		Russia, Arkhangelsk Oblast
*Avenella flexuosa* (L.) Drejer		OR907748–OR907856	Great Britain
*Avenella flexuosa* (L.) Drejer		PQ283993–PQ284022	Russia, Leningrad Oblast

**Table 2:** Major ribotypes of the studied species.

Species	Total Number of Reads	Ribotype Symbol	Number of Reads	% from the Total Number of the Reads
*Deschampsia antarctica*	23,239	An1	7063	30
		An2	5277	23
*Deschampsia brevifolia*	13,928	C1	1804	13
		P2/B2	1326	10
		S2	1162	7
		B4	1016	7
*Deschampsia cespitosa*, USA	18,111	CA1	4988	28
		CA2	3259	18
		C1	1309	13
*Deschampsia cespitosa*, Great Britain	22,596	C1	5451	24
		C2	3032	13
		C3	2926	13
*Deschampsia pamirica*	16,025	S3	2821	18
		P2/B2	1175	7
		P	1154	7
*Deschampsia parvula*	15,241	Pa1	5379	35
		Pa2	1677	11
		Pa3	1034	7
*Deschampsia sukatschewii*	20,619	S1	3511	17
		S2	1594	8
		S3	1582	8
*Deschampsia* sp. Alt 15-434	10,044	S1	2237	22
		S3	1731	17
*Avenella flexuosa*	20,474	Fl	8745	43

## Data Availability

All data generated or analyzed during this study are contained within the article.
